# Real-world use of PSMA imaging in metastatic castration-sensitive prostate cancer: Findings from the CAPRI-3 registry

**DOI:** 10.1007/s00259-026-08050-7

**Published:** 2026-07-10

**Authors:** K. J. M. van der Velden, D. Bosch, Z. Vassilev, A. M. Bergman, A. C. M. van den Bergh, M. J. van der Doelen, A. J. M. van den Eertwegh, J. Lavalaye, R. J. A. van Moorselaar, D. M. Somford, M. Tascilar, A. Raval, C. A. Uyl-de Groot, M. C. P. Kuppen, I. M. van Oort

**Affiliations:** 1 Department of Urology, Radboud University Medical Center, Geert Grooteplein Zuid 10, PO Box: 9101, 6525 Nijmegen, GA The Netherlands; 2 Bayer Healthcare Pharmaceuticals, Healthcare Pharmaceuticals, Whippany, NJ USA; 3 Department of Medical Oncology, Netherlands Cancer Institute (NKI), Amsterdam, Netherlands; 4 Department of Medical Oncology, Amsterdam University Medical Center, Amsterdam, Netherlands; 5 Department of Radiation Oncology, Antoni Van Leeuwenhoek (AVL), Amsterdam, Netherlands; 6 Department of Urology, Zuyderland Medical Center, Heerlen, Netherlands; 7 Department of Medical Oncology, Amsterdam UMC, VU University Medical Centre, Cancer Centre Amsterdam, Amsterdam, Netherlands; 8 Department of Nuclear Medicine, St. Antonius Hospital, Nieuwegein, Netherlands; 9 Department of Urology, Amsterdam University Medical Center, Amsterdam, Netherlands; 10 Department of Urology, Canisius Wilhelmina Hospital (CWZ), Nijmegen, Netherlands; 11 Department of Medical Oncology, Isala Hospital, Zwolle, Netherlands; 12 Erasmus School of Health Policy and Management, Erasmus University Rotterdam, Rotterdam, Netherlands; 13 Department of Radiation Oncology (Maastro), GROW Research Institute for Oncology and Reproduction, Maastricht University Medical Centre+, Maastricht, Netherlands; 14 Department of Urology, University Medical Center Groningen (UMCG), Groningen, Netherlands

**Keywords:** Imaging, Computer tomography, PSMA-PET/CT, Metastatic castration-sensitive prostate cancer, Real-world evidence

## Abstract

**Purpose:**

Since 2019, EAU guidelines recommend PSMA-PET/CT for staging high-risk prostate cancer. However, real-world data on its uptake and association with disease characteristics and outcomes in metastatic castration-sensitive prostate cancer (mCSPC) remains limited. We evaluated real-world PSMA-PET/CT use versus conventional imaging (CI).

**Methods:**

This retrospective study included 3,644 mCSPC patients (2016–2021) from 19 hospitals in the Dutch CAPRI-3 registry. Staging within 90 days of diagnosis was classified as CI-only (CT, bone scintigraphy, and/or MRI) or PSMA-PET (± CI).

**Results:**

Most patients were staged with CI-only (63%), but PSMA-PET use rose from 8% in 2016 to 62% in 2021. PSMA-PET-staged patients were younger (72 vs. 76 years) and had significantly lower tumour burden (27% vs. 45% high-volume), more nodal-only disease, and were more likely to have metachronous presentation (29% vs. 22%, all *P* < 0.001). In the PSMA-PET group, 42% received ADT plus targeted therapy and 23% ADT monotherapy (vs. 45% ADT monotherapy in CI-only). Median overall survival was significantly longer in the PSMA-PET cohort (78.3 vs. 40.0 months).

**Conclusion:**

There has been an increasing trend toward the use of PSMA PET/CT for staging mCSPC in recent years, identifying a population with more favourable disease characteristics and a more favourable prognosis. This shift in patient characteristics, driven by stage migration (the Will Rogers phenomenon), influences clinical management by enabling more refined patient selection for conservative or targeted therapies. Consequently, accounting for lead-time and selection bias is essential when comparing survival outcomes in the PSMA PET/CT era.

**Supplementary Information:**

The online version contains supplementary material available at https://doi.org/10.1007/s00259-026-08050-7.

## Introduction

The introduction of prostate-specific membrane antigen (PSMA)–based imaging with PET/CT has significantly impacted the diagnostic landscape for prostate cancer. By offering superior accuracy as compared to conventional imaging (CI), typically including computed tomography (CT) and bone scintigraphy or Magnetic Resonance Imaging (MRI), PSMA-PET/CT has become the standard for detecting nodal and distant metastases [1–4]. In the landmark ProPSMA trial, PSMA-PET/CT demonstrated a 27% higher absolute accuracy than CI (92% vs 65%), with significantly higher sensitivity (85% vs 38%) and superior specificity (98% vs 91%) for identifying metastatic disease [1]. This diagnostic shift has induced significant stage migration in patients with metastatic castration-sensitive prostate cancer (mCSPC), with 20–40% of patients reclassified upon PSMA-PET/CT imaging, directly impacting treatment selection and outcomes [5, 6].

While formal approval by the European Medicines Agency (EMA) and the U.S. Food and Drug Administration came later in 2020, PSMA-PET/CT was already used through research protocols, early-access programs, and off-label indications. Since its approval, it has been rapidly integrated into international guidelines, particularly for patients with suspected prostate cancer metastases or biochemical recurrence [7–11]. Although previous studies indicate that PSMA-PET/CT alters management in nearly one-third of cases [8, 12, 13], there remains limited evidence regarding how this imaging modality influences real-world systemic treatment selection in the mCSPC setting.

To address this gap, this study uses data from the Dutch CAPRI-3 registry to evaluate the integration of PSMA-PET/CT into clinical practice. Specifically, we compared to patient demographics, clinical characteristics, and the selection of systemic therapies between patients staged with conventional imaging versus those staged with PSMA-PET/CT.

## Materials and methods

### Study design and setting

CAPRI-3 is an ongoing observational retrospective multicenter cohort registry collecting data from patients diagnosed with mCSPC between 2016 and 2021 across 19 hospitals in the Netherlands. The study design has been described before and is registered in the Dutch Trial Registry (NTR Trial ID NL9372) [14, 15]. The study protocol was approved by the Medical Research Ethics Committee Oost-Nederland (CMO 2020–6573) and the institutional review boards of all participating centres. Written informed consent was obtained from alive subjects, except for patients who were lost to follow-up.

### Study population

All completed clinical records of patients with mCSPC in the CAPRI-3 registry were eligible. mCSPC was defined by the presence of regional lymph node involvement (N1) or distant metastatic disease (M1a-c) on imaging. This included both synchronous disease (metastases present at initial diagnosis) and metachronous disease (recurrence/progression following prior local therapy). Additionally, to reflect real-world clinical practice, patients were included if palliative androgen deprivation therapy (ADT) was initiated based on a clinical diagnosis of metastatic disease by the treating physician, even in the absence of identifiable imaging reports. All patients were followed until death, lost-to-follow-up or at last December 31, 2023 (database cut-off).

### Data collection

Imaging data were extracted using automated software (CTcue Data Collector v4.7.0) and supplemented by a quality control (QC) audit [15]. The audit revealed that 97.5% of "missing imaging" cases actually had imaging performed (e.g., at referring hospitals) that was not captured by the software due to interoperability issues (e.g., PDF reports). Consequently, patients without identifiable imaging data were included in the baseline descriptive characteristics to maintain a representative real-world cohort but were excluded from comparative analyses to prevent classification bias.

### Imaging classification and volume staging

Following the exclusion of patients with missing imaging data, the remaining cohort (*n* = 3,644) was categorized into two primary groups based on imaging performed within 90 days of mCSPC diagnosis:

(1) conventional imaging only (CI-only) group, i.e. those who underwent CT, bone scintigraphy and/or MRI; (2) PSMA-PET/CT (PSMA-PET) group, i.e. those who underwent PSMA-PET/CT, either alone or combined with CI.

Reflecting real-world Dutch practice, all PSMA-targeted tracers (e.g., ^68^Ga-PSMA-11, ^18^F-DCFPyL, and ^18^F-PSMA-1007) were eligible for inclusion. Scans were interpreted by local nuclear medicine physicians and radiologists as part of routine clinical care according to (inter)national oncological reporting standards (e.g., RECIST v1.1, miTNM) [16, 17]. In accordance with the registry’s real-world design, the definitive staging and interpretation of suspicious lesions, including the distinction between metastatic disease and non-specific uptake, were based on the final conclusion of the local multidisciplinary team meeting.

Disease volume was stratified according to CHAARTED criteria [18]. To ensure consistency across the total cohort, PSMA-PET/CT findings were treated as equivalent to CI findings for volume staging purposes. In cases of discrepancies between imaging modalities, the multidisciplinary team’s final assessment prevailed. As only the multidisciplinary team’s final integrative staging conclusion was recorded, separate pre- and post-PSMA-PET volume classifications were not available. For comparative analyses, patients were further stratified into four subgroups: (1) CI-only low-volume, (2) CI-only high-volume, (3) PSMA-PET low-volume, and (4) PSMA-PET high-volume.

### Outcomes

The primary outcome of this study was to describe imaging utilization trends at mCSPC diagnosis. Secondary outcomes included assessment of systemic treatment patterns and overall survival (OS). OS was defined as the time from mCSPC diagnosis to death due to any cause, as recorded in the electronical health record.

### Statistical analysis

Descriptive statistics were used to summarize data on patient characteristics and the course of disease and treatment. Continuous variables (median/IQR) and categorical variables (frequencies/percentages) were compared using Mann–Whitney U, Kruskal–Wallis, or Chi-square tests. OS was analyzed using Kaplan–Meier curves. Given the substantial baseline imbalances between imaging cohorts, no formal between-group statistical comparison was performed. A two-sided *p* value of < 0.05 was considered statistically significant. Statistical analyses were performed using SPSS 27.0 (IBM Corp., Armonk, NY, USA) and figures were created using RStudio (version 2024.04.2; RStudio Team, 2024).

## Results

### Study population and baseline characteristics

**Fig. 1:**
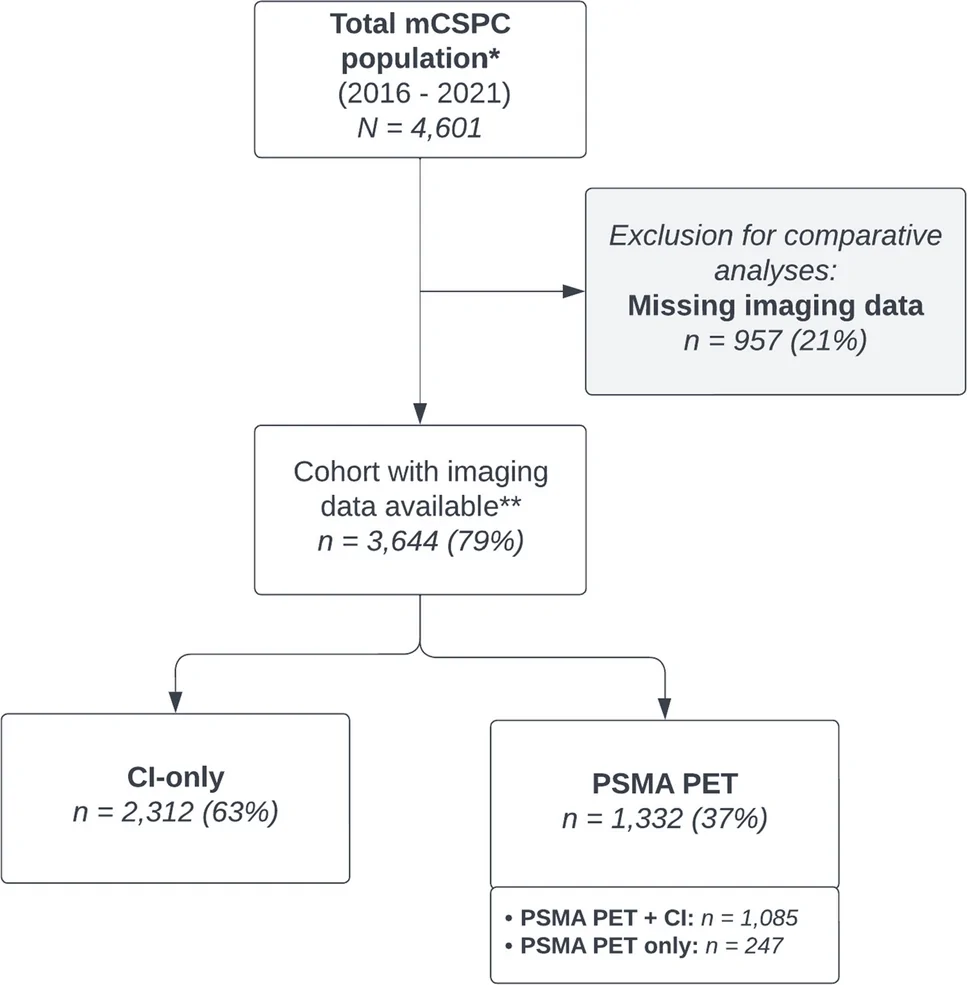
Flowchart of patient selection and subgroups. * Total cohort (*N* = 4,601) was used to describe overall baseline characteristics. ** Comparative analyses for treatment patterns were restricted to patients with available imaging data (*n* = 3,644). *Abbreviations*: mCSPC, metastatic castration-sensitive prostate cancer. CI, conventional imaging. PSMA, PSMA PET/CT

A total of 4,601 mCSPC patients were identified in the CAPRI registry, with a median follow-up of 37.0 months (IQR 20–56 months). 3,644 patients (79%) had available imaging data at diagnosis (Fig. 1). The median age was 74 years (IQR: 68–81 years), and the majority (71%) presented with synchronous metastatic disease. High-volume disease was present in 35% of the total population, based on final multidisciplinary team’s-determined volume classification (Supplementary Table 1).

### Comparison by imaging modality

#### Patient characteristics

Staging was performed with CI-only in 2,312 patients (63%) and PSMA-PET in 1,332 patients (37%). Patients staged with PSMA-PET were significantly younger than those in the CI-only group (median 72 vs. 76 years), presented with a lower rate of Charlson Comorbidity Index (CCI) and a lower Gleason score at initial diagnosis (Table 1). Furthermore, the PSMA-PET group was more likely to present with metachronous disease (29% vs. 22%) and nodal-only involvement (47% vs. 24%, both *p* < 0.001).

**Table 1:** Baseline clinical and disease characteristics compared by imaging modality (conventional imaging vs. PSMA PET/CT)

	CI-only *n* = 2,312	PSMA PET* n = 1,332	*P*-value**
Age (years)	76 (69–82)	72 (66–77)	** < 0.001**
Age-adjusted CCI			
0–2	376 (16)	321 (24)	** < 0.001**
3–4	947 (41)	604 (45)	
5–6	611 (26)	283 (21)	
> 6	378 (16)	124 (9)	
ECOG PS^A^			
0–1	147 (6)	135 (10)	** < 0.001**
2	34 (2)	13 (1)	
≥ 3	31 (1)	1 (< 1)	
Unknown	2,100 (91)	1,183 (89)	
Gleason score at initial diagnosis			
≤ 7	606 (25)	546 (41)	** < 0.001**
8	498 (17)	239 (18)	
9–10	900 (37)	470 (35)	
Unknown	408 (18)	77 (6)	
Metastases at mCSPC diagnosis			
Nodal only	556 (24)	610 (47)	** < 0.001**
Bone metastasis ± nodal disease	1,464 (63)	629 (47)	
Visceral metastasis ± nodal disease	34 (2)	26 (2)	
Both bone and visceral ± nodal disease	126 (5)	57 (4)	
No diagnosed metastases	15 (1)	5 (< 1)	
Unknown	2 (< 1)	5 (< 1)	
Number of bone metastatic sites	1,590 (100)	686 (100)	** < 0.001**
1 site	198 (13)	176 (26)	
2–3 sites	205 (13)	136 (20)	
≥ 4 sites	1,087 (68)	349 (51)	
Unknown	100 (6)	25 (4)	
Disease Volume (CHAARTED)			
High	1,033 (45)	360 (27)	** < 0.001**
Low	646 (28)	879 (66)	
Unknown	633 (27)	93 (7)	
PSA (ng/mL)	85.1 (22–340)	23.5 (7–69)	** < 0.001**
No measurement done	434 (19)	300 (23)	
ALP (U/L)	130 (84–228)	87 (70–116)	** < 0.001**
No measurement done	1,143 (49)	965 (72)	
LDH (U/L)	229 (189–293)	207 (180–246)	
No measurement done	1,454 (63)	1,098 (82)	** < 0.001**
Hb (mmol/L)	8.1 (7.1–8.9)	8.6 (7.6–9.2)	**0.026**
No measurement done	782 (34)	741 (56)	
Time from diagnosis to mCSPC (months)	0.2 (0.0–3)	0.8 (0.1–19)	** < 0.001**
Synchronous mCSPC^+^	1,806 (78)	947 (71)	
Metachronous mCSPC^++^	506 (22)	385 (29)	
Metastatic distribution at initial diagnosis (synchronous only)			
N1M0	262 (15)	267 (28)	** < 0.001**
M1a	54 (3)	66 (7)	
M1b	2 (< 1)	3 (< 1)	
M1c	1,325 (73)	520 (55)	
M1 general	0 (0)	0 (0)	
Unknown	163 (9)	91 (10)	
Local treatments			
EBRT	616 (27)	706 (53)	** < 0.001**
Radical prostatectomy	240 (10)	274 (21)	** < 0.001**

Data are presented as median (interquartile range), or n (%). All laboratory parameters are measured in 6 weeks prior to 1 week to mCSPC diagnosis. *CI* conventional imaging; *PSMA PET* PSMA PET/CT; *CCI* charlson comorbidity index; *ECOG PS* eastern cooperative group performance score; *ISUP* international society of urological pathology; *PSA* prostate specific antigen; *ALP* alkaline phosphatase; *LDH* lactate dehydrogenase; *Hb* haemoglobin; *mCSPC* metastatic castration-sensitive prostate cancer; *EBRT* external beam radiation therapy * Includes patients staged with PSMA PET/CT alone (5%) or PSMA PET/CT in combination with CI (24%). ** P-value refers to the comparison between the CI-only and PSMA PET groups.^+^ Synchronous: metastases ≤ 6 months from initial diagnosis. ^++^ Metachronous: metastases > 6 months

#### Disease burden and modality-specific distribution

Analysis of disease burden by imaging strategy revealed a distinct shift in stage distribution. Within the PSMA-PET-staged cohort, 1,085 patients (81%) underwent both CI and PSMA-PET/CT, while 247 (19%) received PSMA-PET/CT alone (Supplementary Table 2). The prevalence of high-volume disease was highest in the CI-only group (45%), followed by the dual-modality (29%) and PSMA-PET-only (17%) subgroups. Notably, the combined imaging approach significantly reduced the proportion of patients with unknown volume status (6% vs. 27% in the CI-only group).

#### Metastatic patterns and biochemical markers

In synchronous mCSPC, the CI-only group presented with more advanced disease stages. Conversely, the PSMA-PET group showed a higher detection of regional (N1M0: 28% vs. 15%) and non-regional nodal involvement (M1a: 7% vs. 3%, *p* < 0.001).

Across the total cohort, high volume disease was nearly twice as prevalent in the CI-only group (45% vs. 27%). This was reflected by a higher frequency of bone metastases (63% vs. 47%) and a greater skeletal tumour burden (≥ 4 lesions: 68% vs. 51%, both *p* < 0.001). The prevalence of visceral metastases was comparable between the CI-only and PSMA-PET cohorts (7% vs. 6%, respectively). These clinical findings were mirrored by biochemical markers; the PSMA-PET group presented with significantly lower median PSA (23.5 vs. 85.1 ng/mL; *p* < 0.001) and ALP levels (87 vs. 130 U/L; *p* < 0.001) than the CI-only cohort. Local treatments were used more often in the PSMA-PET group. Baseline characteristics for the 957 patients (21%) without available imaging data are provided (Supplementary Table 1).

### Imaging utilization trends

**Fig. 2:**
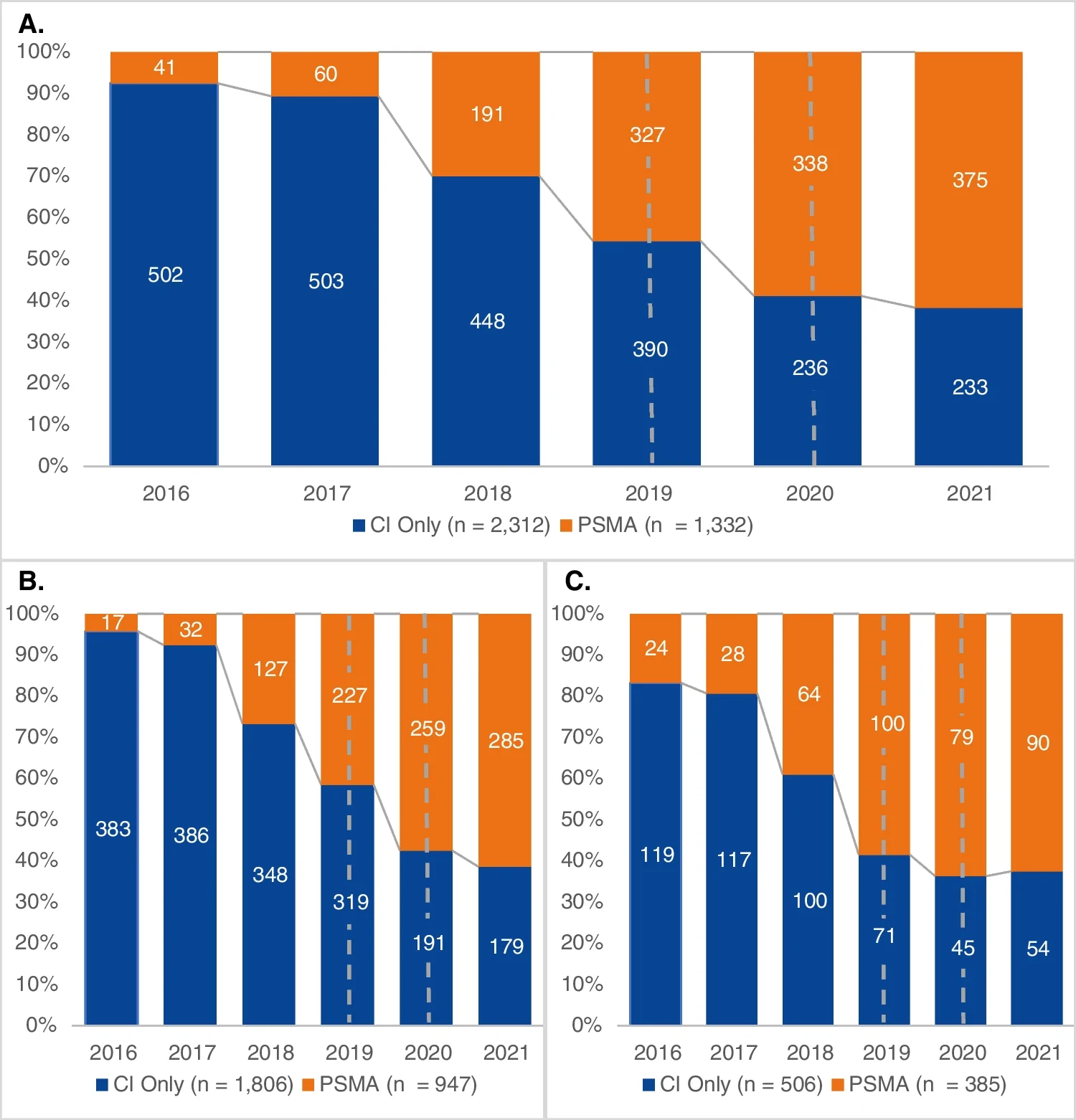
Utilization of imaging modalities at metastatic castration-sensitive prostate cancer diagnosis from 2016 to 2021 for; **A**) Total mCSPC cohort; **B**) Synchronous disease; **C**) Metachronous disease. Vertical dashed lines indicate key milestones: 2019—EAU guideline recommendation for PSMA-PET staging; 2020—Amsterdam consensus panel (February) and EMA approval (late 2020) [8, 10, 11]. *Abbreviations*: CI, conventional imaging. PSMA, PSMA PET/CT

The longitudinal analysis of the diagnostic landscape revealed a rapid shift in clinical practice between 2016 and 2021 (Fig. 2A). The annual proportion of patients staged with CI-only decreased from 92% in 2016 to 38% in 2021. During the same period, the utilization of PSMA-PET/CT increased from 8 to 62%, coinciding with the 2019 EAU guideline recommendation and the 2020 Amsterdam EAU/EANM consensus panel and EMA approval [8, 10, 11]. PSMA-PET adoption differed markedly by disease state (Fig. 2B, 2C). In metachronous disease, utilization increased from 17% in 2016 to 63% in 2021, with majority PSMA-PET staging achieved by 2019. In synchronous disease, adoption was initially slower (4% in 2016) but reached comparable levels by 2021 (61%).

### Treatment patterns

**Fig. 3:**
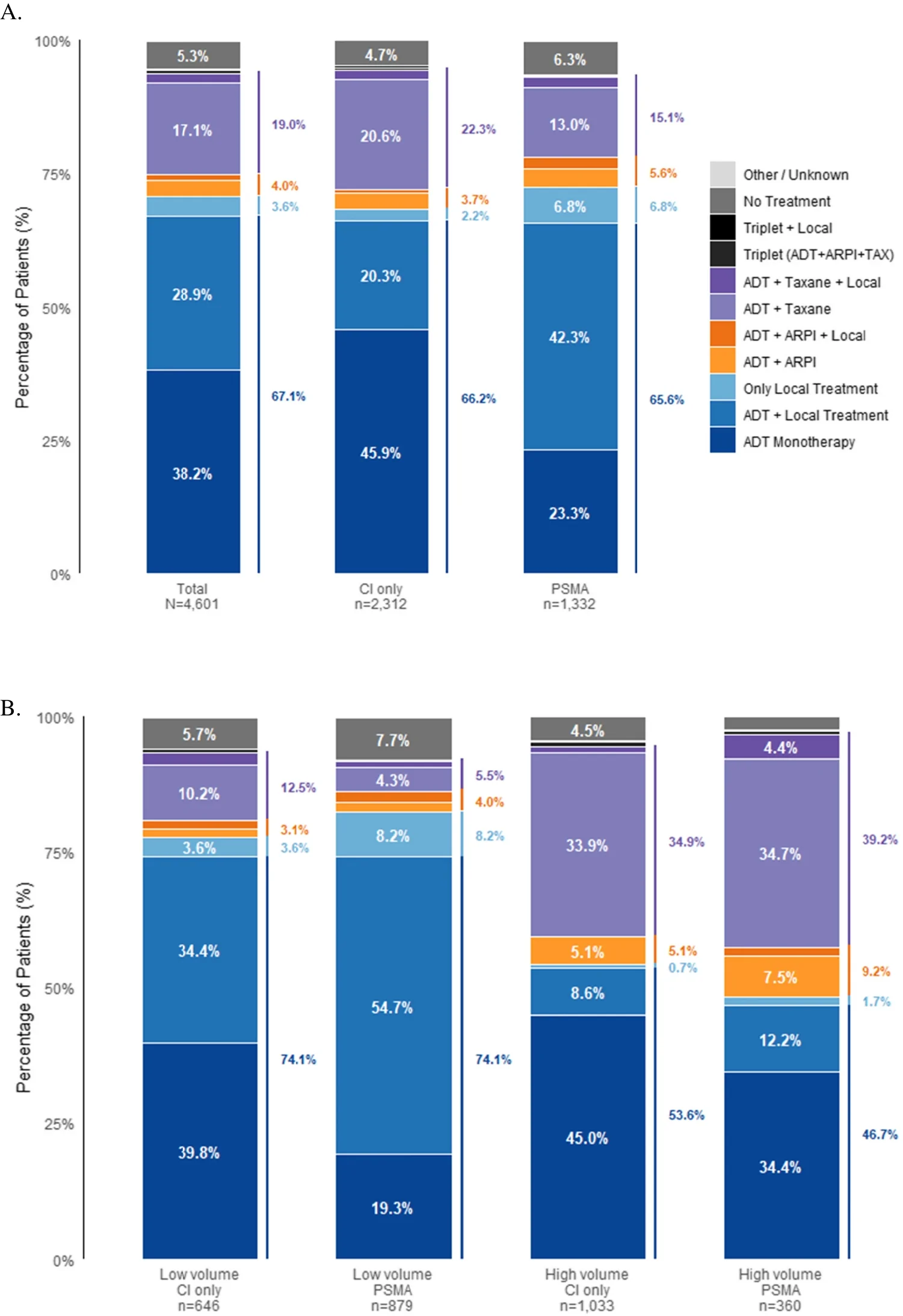
Treatment patterns (**A**) among total cohort (*N* = 4,601) and imaging subgroups; (**B**) among imaging subgroups divided per disease volume. Percentages within the bars represent individual treatment modalities, with the brackets to the right categorizing therapies into clinically relevant groups (e.g., systemic ADT-based regimens, intensified ARPI or taxane-based combinations, and local treatment alone). Local treatment refers to either external beam radiation therapy or radical prostatectomy. *Abbreviations*: ADT, androgen deprivation therapy. ARPI, androgen receptor pathway inhibitor. CI, conventional imaging. PSMA, PSMA-PET/CT

Between 2016 and 2021, ADT monotherapy remained the most prevalent systemic treatment strategy, together with multimodal trajectories involving ADT and local therapy (external beam radiation therapy (EBRT) or radical prostatectomy (RP)) administered to 67.1% of the cohort. Among intensified treatment regimens, taxane-based chemotherapy was the most frequent (19.0%), followed by androgen receptor pathway inhibitors (ARPIs; 4.0%). Only local treatment without systemic therapy was administered to 3.6% of patients (Fig. 3A), predominantly involving those with regional nodal involvement only (N1M0) at diagnosis.

While the overall proportion of ADT-based therapy was comparable (66.2% for CI vs. 65.6% for PSMA-PET), the PSMA-PET-staged group showed a different distribution, with more frequent use of ADT combined with local treatment (42.3% vs. 20.3% in the CI-only group). Furthermore, taxane-based intensification was more frequent in the CI-only group (22.3% vs. 15.1%), whereas ARPI use (5.6% vs. 3.7%), only local treatment (6.8% vs. 2.2%), and no treatment (6.3% vs. 4.67%) were more common in the PSMA-PET cohort. Stratification by disease state confirmed that these treatment shifts were most clearly demonstrated in synchronous disease, where ADT monotherapy was less frequent in PSMA-PET-staged patients (27% vs. 50%) and ADT combined with local therapy was more common (39% vs. 12%), independent of the higher proportion of metachronous patients in the PSMA-PET cohort (Supplementary Table 3).

### Volume and stage migration

Stratification by disease volume further clarified these observed distributions (Fig. 3B). For low-volume disease, PSMA-PET use was associated with a significantly higher utilization of local therapies (62.9% vs. 38.0% for CI-only). Within this subgroup, treatment was most frequently comprised of local therapy combined with ADT (54.7%). In the high-volume setting, both cohorts favoured systemic intensification; however, this trend was more pronounced following PSMA-PET staging. Intensified systemic strategies reached 48.4% in the PSMA-PET cohort (39.2% taxane, 9.2% ARPI) compared to 40.0% in the CI-only cohort (34.9% taxane, 5.1% ARPI). Correspondingly, the reliance on ADT monotherapy decreased from 45.0% in the CI-only group to 34.4% in the PSMA-PET group.

To better understand the observed treatment shifts, we performed an exploratory analysis of baseline characteristics stratified by imaging modality and disease volume (Supplementary Table 4). In the low-volume cohort, patients staged with PSMA-PET presented with a higher prevalence of nodal-only disease (65% vs. 55%) and lower median PSA levels (17 vs. 25 ng/mL) compared to those in the CI-only low-volume cohort. The PSMA-PET high-volume subgroup exhibited a significantly lower biochemical tumour burden, with a four-fold lower median PSA (61 vs. 245 ng/mL) and two-fold lower ALP (109 vs. 203 U/L) compared to the CI-only high-volume subgroup.

### Overall survival

The median OS for the entire cohort was 49.8 months (95%-CI: 23.2 – not estimated (NE)). Median OS was 78.3 months (95%-CI: NE–NE) in the PSMA-PET cohort and 40.0 months (95%-CI: 37.4–42.7) in the CI-only cohort. These findings should be interpreted with caution given the shorter follow-up in the PSMA-PET cohort (69% censored), reflecting its more recent adoption.

## Discussion

This real-world, multicenter analysis from the CAPRI registry describes evolving imaging patterns at mCSPC diagnosis. To our knowledge, representing the largest unselected population to date, these data reflect routine clinical practice between 2016 and 2021, during which PSMA-PET/CT utilization rose from 8 to 62%, while conventional imaging (CI) declined from 92 to 38%. This shift induced stage migration (Will Rogers phenomenon), whereby PSMA-PET's superior sensitivity identifies occult M1 disease in patients previously classified as M0, resulting in more favourable characteristics and longer survival in the PSMA-PET-staged cohort.

The increasing reliance on PSMA-PET/CT in our cohort likely reflects a combination of growing clinical evidence and evolving guidelines and regulatory milestones [7–10]. PSMA-PET utilization rose steadily from 2016 onward, with adoption accelerating around the 2019 EAU guideline recommendation and 2020 Amsterdam EAU/EANM consensus panel and EMA approval [8, 10, 11], suggesting these milestones reinforced rather than initiated the shift toward PSMA-PET staging in routine practice. Adoption was observed across both synchronous and metachronous disease states, though marginally earlier in the latter, likely reflecting pre-existing familiarity with PSMA-PET in the biochemical recurrence setting. Beyond adoption patterns, PSMA-PET-staged patients represent a “ lower-risk” metastatic cohort; they were generally younger, had fewer comorbidities, and presented with lower PSA/ALP levels and higher rates of metachronous, nodal-only, and low-volume disease. This disparity likely reflects a profound selection bias; clinicians may prioritize conventional imaging as a cost-effective means to confirm widespread metastatic disease in patients with a high clinical suspicion (e.g., symptomatic bone pain or very high PSA), while using PSMA-PET/CT in "fitter" patients with low-burden disease to precisely map metastases and guide treatment intensification or metastasis-directed therapy (MDT) [1–6].

Beyond clinician selection, the superior sensitivity of PSMA-PET/CT facilitates technical stage migration [19, 20]. By identifying metastases in patients previously classified as M0, the imaging paradoxically "downstages" the average disease burden of the mCSPC population by incorporating those with lower tumour volumes. Consequently, this stage migration likely results in more favourable clinical characteristics within the remaining M0 population as well, although this remains outside the scope of the CAPRI registry. This precision may optimize resource allocation by sparing patients futile intensive local interventions and facilitating earlier stratification into systemic pathways.

However, an important caveat is that CHAARTED volume definitions were not designed for PSMA-PET imaging. The prognostic equivalence of PSMA-PET-derived versus CI-derived volume classification remains uncertain [5, 21, 22], and PSMA-PET-derived tumor burden may provide improved prognostic stratification beyond conventional criteria. This suggests that novel PSMA-PET-specific volume definitions may be necessary for optimal patient selection in future trials.

Consistent with clinical rationale, the utility of PSMA-PET/CT in our cohort extends beyond the identification of low-volume disease. While the prevalence of visceral metastases remained comparable between the groups (6% vs. 7%), the use of PSMA-PET/CT in this favourable-prognosis cohort likely reflects its superior negative predictive value. This allows clinicians to definitively rule out visceral involvement before committing to treatment intensification or MDT [23, 24]. Furthermore, early PSMA-PET/CT adoption establishes a critical baseline for future precision strategies, such as the potential migration of Lutetium-177-PSMA or Radium-223 into earlier disease stages [25–27].

Despite increased diagnostic precision, ADT-based regimens (monotherapy or combined with local treatment) remained dominant (67.1%), reflecting the known delay in the real-world adoption of treatment intensification [10, 28, 29]. However, the integration of PSMA-PET/CT was associated with a distinct shift in treatment distribution when stratified by disease volume.

In low-volume disease, PSMA-PET-staged patients more frequently received ADT plus local therapy, local-only therapy or even deferred therapy, likely due to the higher prevalence of nodal-only disease (N1/M1a) and lower median PSA levels (17 vs. 25 ng/mL) in the PSMA-PET group. This suggests that PSMA-PET identifies metastatic disease at an earlier disease stage, allowing for more targeted or de-escalated management strategies before widespread progression occurs. A similar trend was observed in the high-volume setting. Despite being classified as "high-volume," the PSMA-PET subgroup exhibited a significantly lower biological burden (PSA 61 vs. 245 ng/mL and ALP 109 vs. 203 U/L compared to the CI-only cohort). This earlier detection, combined with a younger patient age (73 vs. 77 years), likely lowered the threshold for systemic intensification (48% vs. 40%). The observed shift from taxanes (predominant in the CI-only group) to ARPIs (predominant in the PSMA-PET group) likely reflects "era bias", in accordance with updated guidelines prioritizing ARPIs during the period of PSMA-PET/CT adoption [10, 18, 29–31]. Stratification by disease state confirmed that these treatment shifts were independently present in synchronous disease, arguing against confounding by the higher proportion of metachronous patients in the PSMA-PET cohort.

Importantly, these "newly defined" PSMA-PET -detected patients are not represented in the landmark randomized controlled trials that established current standards of care. This creates a potential hazard when extrapolating trial results to a population staged with superior sensitivity. It remains an open question whether the survival benefits observed in CI-staged trial populations can be proportionally translated to patients with PET-only disease, or if this leads to over-treatment of a biologically more indolent disease state. Furthermore, PSMA-PET/CT is not a "gold standard" without flaws; its limitations, including non-specific uptake, PSMA-negative lesions, and suboptimal detection of certain liver metastases, must be considered in clinical decision-making [32].

The higher descriptive median OS in the PSMA-PET group (78.3 vs. 40.0 months) should not be interpreted as a survival benefit attributable to PSMA-PET staging; rather, this difference likely reflects a combination of lead-time bias, due to earlier detection in the disease trajectory, and stage migration. Furthermore, selection bias may play a role, as younger, fitter patients are more likely to be referred for advanced imaging. Recent evidence suggests that patients with metastatic disease detectable only through advanced molecular imaging have a more indolent clinical course compared to those with disease clearly visible on standard conventional imaging [33]. In the biochemical recurrent setting, 84% of patients with no evidence of disease on CI had metastases visible on PSMA-PET/CT [34]. While we cannot definitively classify our cases as 'PET-only' without head-to-head conventional imaging for every patient, the high proportion of low-volume and nodal-only disease in our PSMA-PET cohort suggests a shift towards this more favourable disease spectrum.

This study has limitations inherent to retrospective registry data. First, data interoperability issues led to an underrepresentation of imaging procedures. While this may introduce selection bias, baseline profiles of these 'unclassified' patients are provided to ensure cohort transparency. Second, we lacked the explicit clinical rationale for imaging choices. Specifically, we could not consistently distinguish between standard-of-care and trial-related scans across all centers. Therefore, we omitted this variable to maintain the integrity and representativeness of our national real-world cohort. While this limits our ability to isolate individual physician preference from institutional protocols, it provides a pragmatic reflection of contemporary clinical practice. Lastly, although the CAPRI registry captures radiotherapy for bone metastases, the lack of documented treatment intent prevents a definitive distinction between palliative EBRT and MDT. Nevertheless, the strength of this study lies in its large, unselected, real-world cohort, reflecting routine clinical decision making and real-world uptake of imaging modalities.

## Conclusions

PSMA-PET/CT utilization in mCSPC has increased rapidly, leading to profound stage migration and ostensible survival benefits driven by selection bias. As we transition away from conventional imaging techniques, it is essential to evaluate how this increased diagnostic accuracy can be translated into optimized, evidence-based treatment strategies. Our findings highlight the emergence of a distinct patient population, younger, with lower disease burden and higher rates of nodal-only disease, for whom optimal treatment strategies remain to be defined. Whether this shift in disease spectrum may ultimately support treatment de-escalation for PET-only disease represents an interesting hypothesis that requires prospective validation before clinical implementation.

## Supplementary Information

Below is the link to the electronic supplementary material.

Supplementary file1 (DOCX 25 KB)

Supplementary file2 (DOCX 16 KB)

Supplementary file3 (DOCX 17 KB)

Supplementary file4 (DOCX 21 KB)

## Data Availability

The datasets generated during and/or analysed during the current study are available from the corresponding author on reasonable request.
