# Supplementary material for: Real-world use of PSMA imaging in metastatic castration-sensitive prostate cancer: Findings from the CAPRI-3 registry
Source: Eur J Nucl Med Mol Imaging. 2026 Jul 10;53(12):7017–27. doi: 10.1007/s00259-026-08050-7 (PMC13633343; doi:10.1007/s00259-026-08050-7)
Supplement: Supplementary file 1 [file 259_2026_8050_MOESM1_ESM.docx]

**Supplementary Table 1.** Baseline Clinical and Disease Characteristics of the Total mCSPC Cohort and Subgroups (Conventional Imaging vs. PSMA PET/CT vs. “No Imaging”).

|  | **Total**  N = 4,601 | **CI-only**  n = 2,312 | **PSMA PET*** n = 1,332 | **Missing Imaging Data**  n = 957 |
| --- | --- | --- | --- | --- |
| Age (years) | 74 (68 – 81) | 76 (69 – 82) | 72 (66 – 77) | 74 (67 – 81) |
| Age-adjusted CCI  0-2  3-4  5-6  > 6 | 1 (1 – 2)  913 (20)  1,954 (43)  1,119 (24)  615 (13) | 376 (16)  947 (41)  611 (26)  378 (16) | 321 (24)  604 (45)  283 (21)  124 (9) | 216 (23)  403 (42)  225 (24)  113 (12) |
| ECOG PS^A^   0-1   2  ≥ 3  Unknown | 319 (7)  54 (1)  36 (<1)  4,192 (91) | 147 (6)  34 (2)  31 (1)  2,100 (91) | 135 (10)  13 (1)  1 (<1)  1,183 (89) | 37 (4)  13 (1)  4 (<1)  909 (95) |
| Gleason score at initial diagnosis  ≤ 7 (ISUP 1-3)  8 (ISUP 4)  9-10 (ISUP 5)  Unknown | 1,518 (33)  828 (18)  1,651 (36)  604 (13) | 606 (25)  498 (17)  900 (37)  408 (18) | 546 (41)  239 (18)  470 (35)  77 (6) | 375 (39)  191 (20)  281 (29)  119 (12) |
| Metastases at mCSPC diagnosis**  Nodal only  Bone metastasis +/- nodal disease  Visceral metastasis +/- nodal disease  Both bone and visceral +/- nodal disease  No diagnosed metastases  Unknown | 1,463 (32)  2,482 (54)  70 (2)  215 (5)  25 (1)  343 (8) | 556 (24)  1,464 (63)  34 (2)  126 (5)  15 (1)  2 (<1) | 610 (47)  629 (47)  26 (2)  57 (4)  5 (<1)  5 (<1) | 297 (31)  389 (41)  10 (1)  32 (3)  5 (1)  224 (23) |
| Number of bone metastatic sites  1 site  2–3 sites  ≥ 4 sites  Unknown | 2,697 (100)  448 (17)  398 (15)  1,647 (61)  204 (8) | 1,590 (100)  198 (13)  205 (13)  1,087 (68)  100 (6) | 686 (100)  176 (26)  136 (20)  349 (51)  25 (4) | 421 (100)  74 (18)  57 (14)  211 (50)  79 (19) |
| Volume of disease according to CHAARTED criteria ^(1)^  High volume  Low volume  Unknown | 1,600 (35)  1,854 (40)  1,147 (25) | 1,033 (45)  646 (28)  633 (27) | 360 (27)  879 (66)  93 (7) | 207 (22)  329 (34)  421 (44) |
| PSA (ng/mL)  No measurement done | 44 (12 – 190)  1,174 (26) | 85.1 (22 – 340)  434 (19) | 23.5 (7 – 69)  300 (23) | 27.9 (9 – 95)  440 (46) |
| ALP (U/L)  No measurement done | 108 (77 – 286)  3,383 (74) | 130 (84 – 228)  1,143 (49) | 87 (70 – 116)  965 (72) | 87 (66 – 150)  731 (76) |
| LDH (U/L)  No measurement done | 221 (186 – 286)  3,383 (74) | 229 (189 – 293)  1,454 (63) | 207 (180 – 246)  1,098 (82) | 206 (180 – 259)  831 (87) |
| Hb (mmol/L)  No measurement done | 8.2 (7.3 – 9.0)  2,120 (46) | 8.1 (7.1 – 8.9)  782 (34) | 8.6 (7.6 – 9.2)  741 (56) | 8.3 (7.5 –9.0)  597 (62) |
| Time from diagnosis to mCSPC (months)  Synchronous mCSPC ^+^  Metachronous mCSPC ^++^ | 0.6 (0 – 18.8)  3,287 (71)  1,314 (29) | 0.2 (0 – 2.5)  1,806 (78)  506 (22) | 0.8 (0.1 – 19.2)  947 (71)  385 (29) | 2.4 (0 – 55.8)  534 (56)  423 (44) |
| Local treatments  EBRT  Radical prostatectomy | 1,678 (36)  720 (16) | 616 (27)  240 (10) | 706 (53)  274 (21) | 356 (37)  206 (21) |

Data are presented as median (interquartile range), or n (%). *Abbreviations:* CI, conventional imaging. PSMA PET, PSMA PET/CT. CCI, Charlson Comorbidity Index. ECOG PS, Eastern Cooperative Group Performance Score. ISUP, International Society of Urological pathology. PSA, prostate specific antigen. ALP, alkaline phosphatase. LDH, lactate dehydrogenase. Hb, hemoglobin. mCSPC, metastatic castration-sensitive prostate cancer. EBRT, external beam radiation therapy * Includes patients staged with PSMA PET/CT alone (5%) or PSMA PET/CT in combination with CI (24%). ** For the 'no imaging' group, metastatic status was verified through retrospective record review in the absence of current imaging reports. ^+^ Synchronous mCSPC was defined as detection of at least one metastasis within six months from the initial diagnosis. ^++^ Metachronous mCSPC was defined as detection of at least one metastasis ≥ 6 months from the initial diagnosis.
