# Supplementary material for: Real-world use of PSMA imaging in metastatic castration-sensitive prostate cancer: Findings from the CAPRI-3 registry
Source: Eur J Nucl Med Mol Imaging. 2026 Jul 10;53(12):7017–27. doi: 10.1007/s00259-026-08050-7 (PMC13633343; doi:10.1007/s00259-026-08050-7)
Supplement: Supplementary file 2 [file 259_2026_8050_MOESM2_ESM.docx]

**Supplementary Table 2**. Disease Volume according to CHAARTED criteria, stratified by imaging modality (Conventional Imaging only vs. PSMA PET/CT only vs. both CI and PSMA PET/CT).

|  | **CI-only**  n = 2,312 | **PSMA PET/CT only**  n = 247 | **Both CI and PSMA PET/CT** n = 1,085 |
| --- | --- | --- | --- |
| Volume of disease according to CHAARTED criteria  High volume  Low volume  Unknown | 1,033 (45)  646 (28)  633 (27) | 41 (17)  175 (71)  31 (13) | 319 (29)  704 (65)  62 (6) |

Data are presented as or n (%). Totals may not equal 100% due to rounding. *Abbreviations:* CI, conventional imaging.
