# Supplementary material for: Real-world use of PSMA imaging in metastatic castration-sensitive prostate cancer: Findings from the CAPRI-3 registry
Source: Eur J Nucl Med Mol Imaging. 2026 Jul 10;53(12):7017–27. doi: 10.1007/s00259-026-08050-7 (PMC13633343; doi:10.1007/s00259-026-08050-7)
Supplement: Supplementary file 3 [file 259_2026_8050_MOESM3_ESM.docx]

**Supplementary Table 3**. Treatment patterns stratified by disease state and imaging modality

| **Treatment** | **Synchronous  CI-only**  n = 1,806 | **Synchronous  PSMA PET**  n = 947 | **Metachronous  CI-only**  n = 506 | **Metachronous PSMA PET**  N = 385 |
| --- | --- | --- | --- | --- |
| ADT monotherapy  ADT + local therapy (EBRT/RP)*  ADT + taxane  ADT + ARPI  Triplet therapy  Local therapy only  Other  No treatment | 899 (50)  220 (12)  483 (27)  77 (4)  14 (1)  17 (1)  2 (<1)  94 (5) | 259 (27)  371 (39)  180 (19)  55 (6)  4 (<1)  25 (3)  0 (0)  53 (6) | 162 (32)  249 (49)  33 (7)  8 (2)  4 (1)  33 (7)  2 (<1)  15 (3) | 52 (14)  193 (50)  21 (6)  19 (5)  1 (<1)  65 (17)  3 (1)  31 (8) |

Data are presented as or n (%). *Abbreviations:* CI, conventional imaging. PSMA PET, PSMA PET/CT. ADT, androgen deprivation therapy. EBRT, external beam radiation therapy. RP, radical prostatectomy. ARPI, androgen receptor pathway inhibitor. Triplet therapy, ADT + ARPI + chemotherapy. *In metachronous patients, local therapy may include prior curative-intent treatment (RP or EBRT) administered before metastatic diagnosis, as the variable captures all local treatment prior to CRPC regardless of timing relative to metastatic diagnosis. This limitation does not apply to synchronous patients, who by definition had no prior local treatment at metastatic presentation.
